# Epidemiological investigation and pathogenicity of *Streptococcus suis* in eastern China

**DOI:** 10.3389/fmicb.2025.1710390

**Published:** 2026-01-21

**Authors:** Dehong Yang, Jingyu Xu, Meiling Hu, Jinmei Zhu, Baihua Ren, Xianhui Huang, Lianxiang Wang

**Affiliations:** 1 College of Veterinary Medicine, South China Agricultural University, Guangzhou, China; 2 Guangdong Enterprise Key Laboratory for Animal Health and Environmental Control, Wen's Foodstuff Group Co. Ltd, Yunfu, China

**Keywords:** *Streptococcus suis*, epidemiological investigation, serotype, virulence gene, pathogenicity

## Abstract

*Streptococcus suis (S. suis)*, a zoonotic gram-positive bacterium, is the etiological factor for septicemia and pneumonia in humans and pigs and poses a global public health threat. To date, epidemiological data from large-scale investigations of *S. suis* in swine populations across eastern China are still limited. This study investigated the serotypes, virulence genes, and pathogenicity of the isolates from 89 pig farms across 12 regions from 2022 to 2024. The overall infection and isolation rates were 59.59% (851/1728) and 16.1% (137/851), respectively. The infection rate was the highest in Guangdong Province (72.41%) (63/87) and the lowest in Hubei Province (43.75%) (7/16). Suckling piglets, nursery pigs, fattening pigs, and pregnant sows are susceptible to S. suis infection, with infection rates as high as 60%. The infection rates in spring, summer, autumn, and winter were 70.72% (215/304), 60.67% (344/567), 40.62% (132/325), and 68.97% (160/232), respectively. Serotype analysis of 137 isolates revealed increased serotype diversity in coastal provinces, especially in Guangdong, Jiangsu, and Shandong. Serotype 1 was detected in Liaoning. The most common serotype was serotype 2 (30.66%), especially in Guangdong, Guangxi, and Anhui, followed by serotype 7 (21.17%) and serotype 9 (10.95%). Virulence gene analysis revealed that the occurrence of *gdh*, *gapdh*, and *orf2* (>89%) was high, whereas that of 89 k and epf was low (≤ 28.47%). Serotypes 1 and 7 frequently harbored mrp and gdh but often lacked 89 k and epf. Serotype 2 and serotype NT harbored all the tested genes, withlow 89 k occurrence rates. The occurrence rates of sly and epf (≤43.75%) werelow for serotype 9. Animal challenge experiments demonstrated that Serotype 2 induced acute death in Landrace pigs aged 42 days, with a mortality rate of 100%. In contrast, Serotype 7 was associated with low mortality rates (37.5%) and induced mild pathological symptoms, including pneumonia, myocarditis, and yellow effusion in the thoracic cavity. This study provides useful insights for the prevention and control of S. suis infection on pig farms in China.

## 1 Introduction

*S. suis*, a gram-positive bacterium that typically appears in pairs or short chains, can infect various animals, including pigs (Mi et al., 2021), horses (Devriese and Haesebrouck, 1992), dogs, and cats (Salasia et al., 1994), as well as humans (Bamphensin et al., 2021). In pigs, *S. suis* infections cause septicaemia, pneumonia, arthritis, and endocarditis. Moreover, *S. suis* infection can lead to symptoms such as septicemia, skin ulcers, and meningitis (Luque et al., 1998).

*S. suis* was initially classified into 35 serotypes on the basis of the presence of capsular antigens (Liu et al., 2013). However, advances in molecular techniques have refined the classification system. Currently, *S. suis* is divided into 29 classical serotypes (Nomoto et al., 2015). The serotype distribution of *S. suis* exhibits marked regional variation. Serotype 2, the most virulent global strain, accounts for 74.7% of human infections and dominates swine populations in Asia (44.2%) and North America (24.3%), whereas serotype 9 prevails in Europe (61%) (Haas and Grenier, 2018). Notably, South Korea shows an anomalous predominance of serotypes 3/4, and European countries such as Spain and the Netherlands display evolving serotype patterns (9/2/7) (Wisselink et al., 2000). The paucity of data regarding *S. suis* serotype patterns in the swine population of eastern China presents considerable concern and a latent risk to regional disease control efforts, underscoring the urgency of forming an interdisciplinary surveillance network to mitigate this zoonotic threat.

The pathogenicity of *S. suis* is closely related to its virulence factors. The main virulence genes of *S. suis* include *mrp*, *epf*, *sly*, *orf2*, *fbps*, *gdh, gapdh*, and *89 k*, which are involved in the pathogenic process. Among the virulence genes, *mrp*, *epf*, and *sly* are considered the key virulence factors of *S. suis* (Silva et al., 2006). In Eurasian strains, these genes are positively correlated with pathogenicity. The frequent phenotypes of diseased and healthy pigs are *mrp*^+^*epf*^+^*sly*^+^ and *mrp*−*epf*−*sly*−, respectively (Petrocchi-Rilo et al., 2021). However, some highly virulent strains from Canada do not express *mrp*, *epf*, or *sly* (Gottschalk et al., 1998). Additionally, avirulent strains harboring all three of these genes have not been identified (Vecht et al., 1992). *Sly*, which encodes a cytotoxic hemolysin, may enhance pathogenicity by modulating complement deposition and promoting the penetration of *S. suis* into deeper tissues (King et al., 2001). *Epf* serves as a phenotypic marker of virulence (Wisselink et al., 1999). The scarcity of systematic data on virulence gene distributions, dominant phenotypes, and their pathogenic associations—stemming from limited *S. suis* research in eastern China-severely hinders the development of targeted interventions.

Prompted by the 2005 human outbreak in Sichuan, China, *S. suis*, a key zoonotic pathogen, has been closely monitored; however, data on its epidemiology and virulence genotypes in eastern China are sparse. This study, which was conducted from 2022 to 2024 across 89 pig farms in 12 provinces, bridges this knowledge gap by serotyping, virulence genotyping, and pathogenicity assessment of dominant serotypes, thereby providing essential evidence for evidence-based, precise control measures in China’s swine industry.

## 2 Materials and methods

### 2.1 Sample collection

This study collected 1,428 samples from over 89 swine operations, including both individual farmers and large-scale commercial farms (about 5 million pigs) across 12 provinces in China (Anhui (129), Guangdong (87), Guangxi (143), Hainan (68), Hubei (16), Hunan (246), Jiangsu (119), Jiangxi (85), Liaoning (26), Shandong (98), Shanxi (119), and Shaanxi (291)) between 2022 and 2024. The samples included nasopharyngeal swabs, pleural effusion, joint fluid from the legs, lungs, brain tissue, and vaginal pus from diseased piglets, nursery pigs, fattening pigs, and breeding pigs suspected of having *S. suis* infections. These pigs presented symptoms such as fever, swollen joints, and emaciation. Post-mortem examinations revealed septicemia, polyserositis, pneumonia, myocarditis, and hemorrhaging in multiple organs. All sample collections were conducted with the informed consent of the managers of each pig farm.

### 2.2 Bacterial isolation and identification

The samples were inoculated on tryptic soy agar (TSA, Difco Laboratories, Detroit, MI, USA) plates containing 5% bovine serum and incubated aerobically at 37 °C for 48 h. The colonies were selected and cultured further in Todd-Hewitt broth (THB) with shaking at 37 °C for 16 to 18 h. The broth cultures were subjected to polymerase chain reaction (PCR) to identify *S. suis* through amplification of the *gdh* gene (Kerdsin et al., 2012). Positive strains were streaked onto THB plates for colony purification. The purified strains were heated in boiling water for 10 min and centrifuged at 13,000 × g for 10 min. The supernatant was stored at −20 °C until use.

### 2.3 Serotyping

The methods of previous studies were used (Kerdsin et al., 2014; Kerdsin et al., 2012; Liu et al., 2013; Smith et al., 1999). The serotypes of the isolated *S. suis* strains were determined using the primers listed in Table 1 (synthesized by Sangon Biotech (Shanghai) Co., Ltd.). PCR amplification was performed using 2 × Taq Quick-Load Master Mix (CW Biotech, Beijing, China) under the following conditions: 95 °C for 5 min (initial denaturation), followed by 35 cycles of 95 °C for 1 min (denaturation), 56 °C for 1 min (annealing), 72 °C for 1 min (annealing), and 72 °C for 5 min (final extension). Each sample was analyzed in triplicate. The amplicons were analyzed using 2% agarose gel electrophoresis.

**Table 1:** Primers for identifying and serotyping *S. suis*.

Serotype	Sequence (5′–3′)	Annealing temperature (°C)	PCR product sizes (bp)	Gene	Reference
All	F: TTCTGCAGCGTATTCTGTCAAACG	55	695	gdh	Kerdsin et al. (2012)
	R: TGTTCCATGGACAGATAAAGATGG				
1	F: GGCGGTCTAGCAGATGCTCG	56	440	cps1I	Smith et al. (1999)
	R: GCGAACTGTTAGCAATGAC				
2	F: CAAACGCAAGGAATTACGGTATC	56	675	cps2J	Smith et al. (1999)
	R: GAGTATCTAAAGAATGCCTATTG				
3	F: TGGGAGAAGGCAGAAAGTACGAGA	60	1,273	cps3J–cps3K	Kerdsin et al. (2012)
	R: ACCCCCAGAAGAGCCGAAGGA				
4	F: ACTTGGAGTTGTCGGAGTAGTGCT	60	783	cps4M–cps4N	Kerdsin et al. (2012)
	R: ACCGCGATGGATAGGCCGAC				
5	F: TGATGGCGGAGTTTGGGTCGC	60	166	cps5N	Kerdsin et al. (2012)
	R: CGTAACAACCGCCCCAGCCG				
6	F: TACGGTCTCCCTTGCCTGTA	60	325	cps6I	Kerdsin et al. (2014)
	R: AACTCAGCTAGTGCTCCACG				
7	F: GATGATTTATGGCACCCGAGTAAGC	60	150	cps7H	Kerdsin et al. (2012)
	R: AGTCACAATTGCTGGTCCTGACACC				
8	F: ATGGGCGTTGGCGGGAGTTT	60	320	cps8H	Kerdsin et al. (2012)
	R: TTACGGCCCCCATCACGCTG				
9	F: GGGATGATTGCTCGACAGAT	60	300	cps9H	Kerdsin et al. (2012)
	R: CCGAAGTATCTGGGCTACTG				
10	F: TTACGAGGGGATTCTGGGGT	60	153	cps10M	Kerdsin et al. (2014)
	R: CGGGACAACAGATGGAACCT				
11	F: TACAGTGCTTGCAGCCCTAC	60	896	cps11N	Kerdsin et al. (2014)
	R: CGACTTGTCGTGCCCTGAT				
12	F: TGTGGCGATAGGACAACAGG	60	209	cps12J	Kerdsin et al. (2014)
	R: ACCAAGAAGTTTCCGCCTGA				
13	F: CTGGTGCTGCAATTTCGCTT	60	1,135	cps13L	Kerdsin et al. (2014)
	R: GCAGACTAGCTGCAGTTCCA				
14	F: AATCATGGAATAAAGCGGAGTACAG	60	550	cps14J	Kerdsin et al. (2012)
	R: ACAATTGATACGTCAAAATCCTCACC				
15	F: GCAAGAAAGCTTCCGGATGGA	60	274	cps15K	Kerdsin et al. (2014)
	R: CAAGAGAGTGTGCAACCCCA				
16	F: TGGAGGAGCATCTACAGCTCGGAAT	60	202	cps16K	Kerdsin et al. (2012)
	R: TTTGTTTGCTGGAATCTCAGGCACC				
17	F: ACTTGGGTTGGAATGGCGAA	60	906	cps17O	Kerdsin et al. (2014)
	R: ACCACCGAAAGTCAGGTCAC				
18	F: CGGGGCAGTCTTACTCATGG	60	432	cps18N	Kerdsin et al. (2014)
	R: ATGACAGCGAAACGGACAGA				
19	F: AGCAGGGTTGCGTATGGCGG	60	1,024	cps19L	Kerdsin et al. (2012)
	R: ACAAGCACCAGCAAAGACCGCA				
20	F: TAATCGTTGCCTTTGAGCAT	58	938	cps20I	Liu et al. (2013)
	R: CGCTATATAAGGAAACCTCGG				
21	F: GGTGGCAAGGAGAGCAAAGT	60	325	cps21N	Kerdsin et al. (2014)
	R: ACATGGTAAGCCATTGCTGGA				
22	F: AGGATCGGTAAGTTTAGGTACA	58	158	cps22K	Liu et al. (2013)
	R: ATGCAGTAAAACACGAAAACAA				
23	F: TGCTCAACAAACGCAGCAAA	60	454	cps23I	Kerdsin et al. (2014)
	R: TGACTGGTACATCTGCAGCC				
24	F: ACCCGGAAAAACCAGGAGTT	60	500	cps24L	Kerdsin et al. (2014)
	R: ACCAATCAATGCCAAGCGAC				
25	F: GGAGGAGCTGCGGGCTCATA	60	1,211	cps25M–cps25N	Kerdsin et al. (2012)
	R: TGGCCACAACCTGGATGCGTT				
26	F: CAAAATTCCTGGATTAACGCTT	58	315	cps26P	Liu et al. (2013)
	R: CGATCTGAGGACTTATCAAGAA				
27	F: CTACGCCAATCGAAGCCAGA	60	506	cps27K	Kerdsin et al. (2014)
	R: CCAGTAAGAAGCCTGTCGCA				
28	F: GGACTTCGGTACCTTAGCGT	60	865	cps28L	Kerdsin et al. (2014)
	R: CTCCAGCACATTCCCGTACC				
29	F: GTGCGGGCGTTATTTTTGGT	60	435	cps29L	Kerdsin et al. (2014)
	R: AGCCTTGCAACCCATTTCCT				
30	F: CTTTAATTGCTTGCGCCCGT	60	170	cps30I	Kerdsin et al. (2014)
	R: ATTCGGGCTACCCATTGCAG				
31	F: GGAGTGCTCTATGCCACCTT	60	550	cps31L	Kerdsin et al. (2014)
	R: GCATTGCCCCTACAGCAAAC				
33	F: GAGTTGCGACCTATTATTCTCA	58	731	cps33K	Liu et al. (2013)
	R: GAATTGAACAACGACTGCAATA				

### 2.4 Virulence gene detection

Previous methods have been used (Ju et al., 2008; Kerdsin et al., 2012; Pan et al., 2020). The virulence genes in the isolated *S. suis* strains were identified using the primers listed in Table 2 [also synthesized by Sangon Biotech (Shanghai) Co., Ltd.]. The detection method was based on previou*sly* reported PCR protocols targeting the following genes: *gdh*, *fbps*, *sly*, *orf2*, *mrp*, *89 k*, *gapdh*, and *epf*.

**Table 2:** Primers for identifying virulence genes of *S. suis*.

Gene	Sequence (5′-3′)	Annealing temperature (°C)	PCR product sizes (bp)	Reference
gdh	F: TTCTGCAGCGTATTCTGTCAAACG	55	695	Kerdsin et al. (2012)
	R: TGTTCCATGGACAGATAAAGATGG			
fbps	F: ATCGATTCATTTAAATAGGTTCCTGCTCGC	55	2,179	Ju et al. (2008)
	R: GGTACCATTGTTGGTATTTGGACACCAGAA			
sly	F: GCTTTATTGCGTGCTGAC	55	1,099	Ju et al. (2008)
	R: CTGTTCTCCACCACTCCC			
orf2	F: CAAGTGTATGTGGATGGG	56	858	Ju et al., 2008
	R: ATCCAGTTGACACGTGCA			
mrp	F: CAGATGTGGACCGTAGACC	58	316	Ju et al. (2008)
	R: GGATAATCACCAGCAGGAA			
89 k	F: TCGCCACTATGGTATCTGCTTA	56	720	Ju et al., 2008
	R: GATTGTGGACCATGCTCTTTAG			
gapdh	F: CAGTCAAAGCCCGCAACC	58	571	Pan et al. (2020)
	R: CCACCGAAGCCAAGAGGT			
epf	F: GCTACGACGGCCTCAGAAATC	55	626	Ju et al. (2008)
	R: TGGATCAACCACTGGTGTTAC			

### 2.5 Animal pathogenicity experiment

The pathogenicity of isolates SS2-1 (serotype 2) and SS7-1 (serotype 7) was evaluated in 24 healthy Landrace pigs aged 42 days. These pigs tested negative for *S. suis* and other exogenous pathogens, including classical swine fever (CSF), African swine fever (ASF), porcine reproductive and respiratory syndrome (PRRS), and porcine circovirus (PCV). The pigs were randomly divided into the following three groups (seven pigs per group): the SS2, SS7, and control groups. All pigs had free access to water and food.

Pigs in the SS2 and SS7 groups were intraperitoneally injected with 2 mL of 1.0 × 10^6^ CFU of SS2-1 or SS7-1, respectively, while pigs in the control group were injected with an equal volume of sterile phosphate-buffered saline (PBS). Clinical signs and mortality were recorded daily for 14 days post-infection. Dead pigs were immediately necropsied to observe pathological changes, and lung tissues were collected for hematoxylin and eosin (H&E) staining analysis. All experiments were conducted in strict accordance with the Guidelines for the Care and Use of Laboratory Animals and were approved by the Ethics Committee of South China Agricultural University. At the end of the experiment, all surviving pigs were humanely euthanized by intravenous injection of an overdose of pentobarbital sodium following anesthesia to ensure animal welfare.

### 2.6 Data analysis

The analysis and mapping of *S. suis* infection rates, as well as serotype identification, were performed using Office 2021 software. Correlation analysis between serotypes and virulence genes of the isolated strains, along with the generation of related charts, was performed using the online tool https://www.chiplot.online/. The mortality rate analysis and chart creation for the animal experiments were conducted using GraphPad Prism 8 software.

## 3 Results

### 3.1 Detection and infection rate analysis of suspected *S. suis* clinical samples

**Figure 1:**
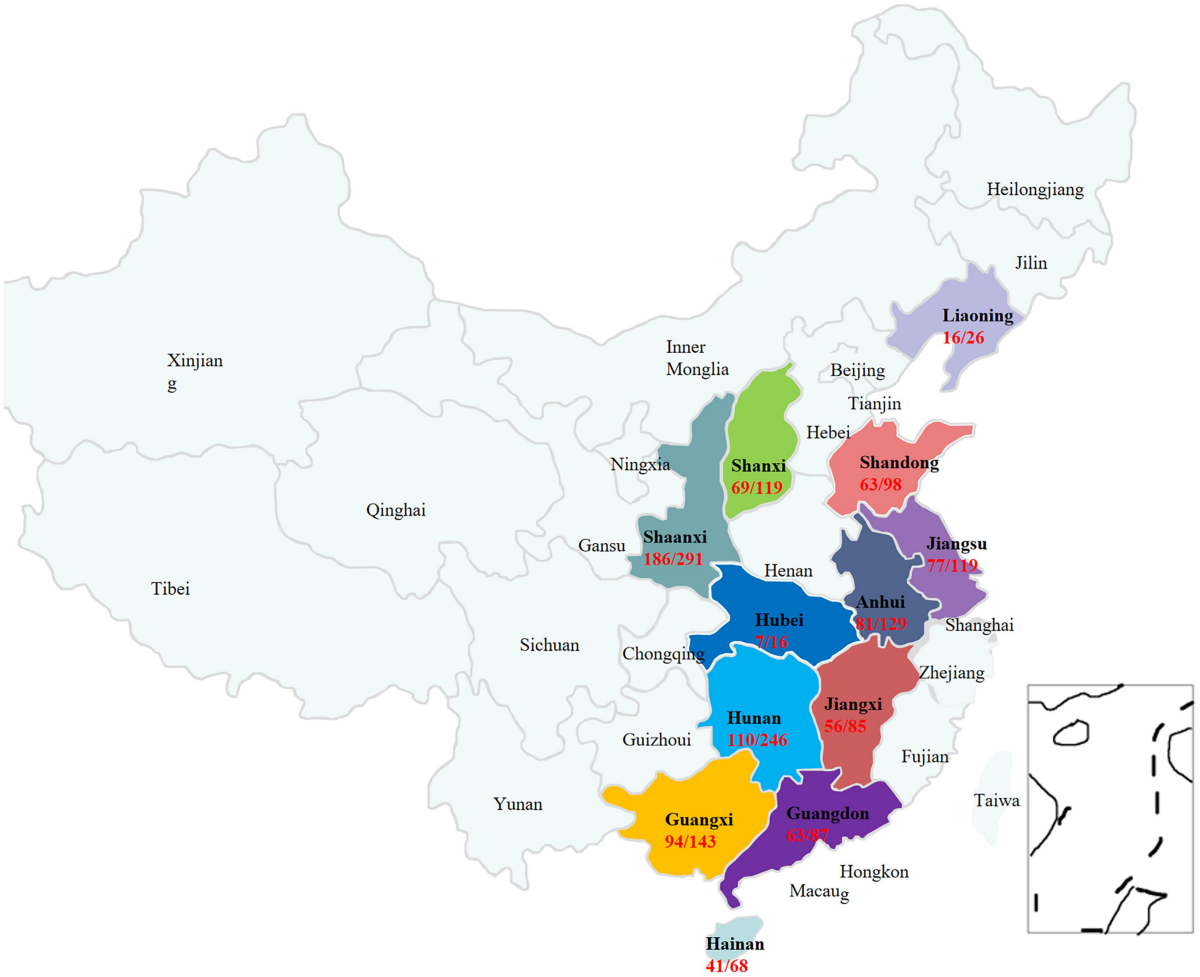
Geographic distribution, sample size, and number of positive samples of suspected *Streptococcus suis* from pigs in 12 regions of China.

To investigate the epidemiological characteristics of *S. suis* in major pig-farming regions of China, 1,428 suspected infection samples were collected from 89 pig farms across 12 provinces and tested using PCR. The distributions of the 12 provinces and the numbers of collected samples and positive test samples are shown in Figure 1.

**Figure 2:**
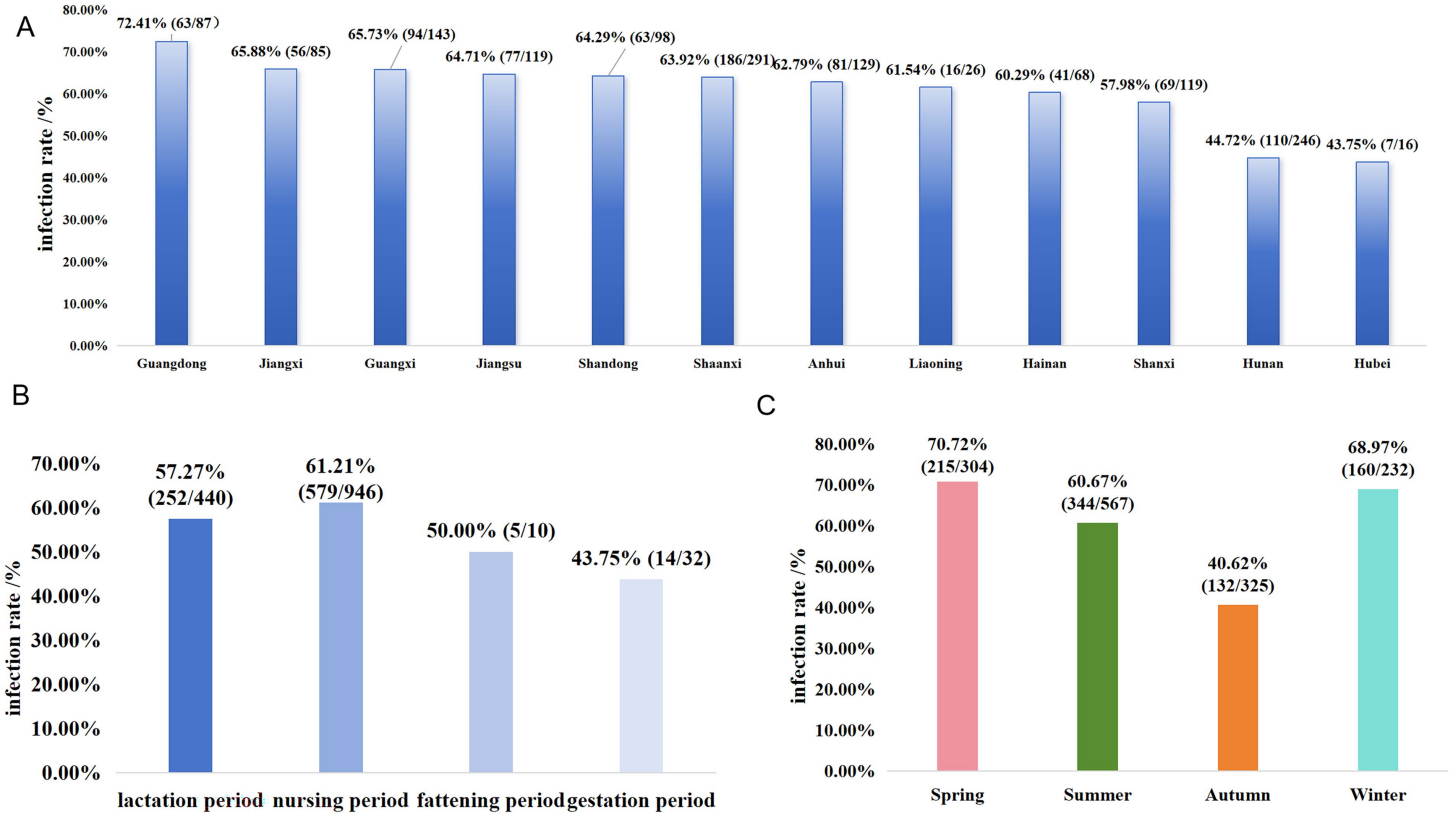
Prevalence of *S. suis* in different regions of China: **(A)** infection rates of *S. suis* in 12 regions of China; **(B)** distribution of *S. suis* infections across different growth stages of pigs; **(C)** seasonal variations in *S. suis* infection rates.

The overall infection and isolation rates were 59.59% (851/1728) and 16.1% (137/851), respectively. The infection rates in different regions were as follows (Figure 2A): Guangdong: 72.41% (63/87); Jiangxi: 65.88% (56/85); Guangxi: 65.73% (94/143); Jiangsu: 64.71% (77/119); Shandong: 64.29% (63/98); Shaanxi: 63.92% (186/291); Anhui: 62.79% (81/129); Liaoning: 61.54% (16/26); Hainan: 60.29% (41/68); Shanxi: 57.98% (69/119); Hunan: 44.72% (110/246); and Hubei: 43.75% (7/16). Additionally, the infection rates in suckling piglets (aged 0–21 days), nursery pigs (aged 21–70 days), growing-finishing pigs (aged 70 days to 6 months, with weights of about 100–120 kilograms), and pregnant sows (sows in the gestation period) were 57.27% (252/440), 61.21% (579/946), 50% (5/10), and 43.75% (14/32), respectively (Figure 2B). Next, the prevalence of *S. suis* infections in spring, summer, autumn, and winter was examined. The infection rates in spring (February–April), summer (May–July), autumn (August–October), and winter (from November to January of the following year) were 70.72% (215/304), 60.67% (344/567), 40.62% (132/325), and 68.97% (160/232), respectively (Figure 2C).

### 3.2 Serotyping of *S. suis*

**Figure 3:**
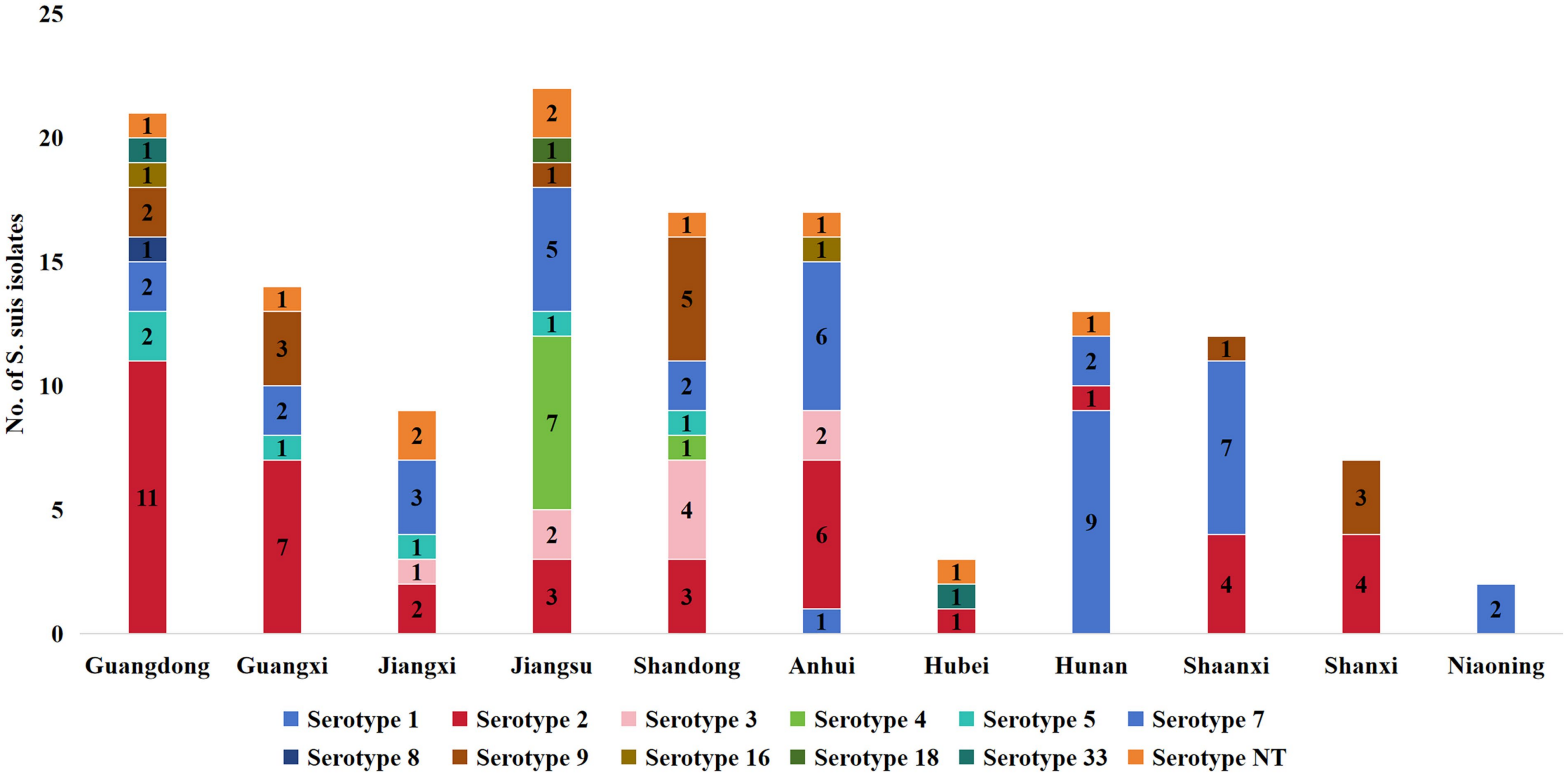
Serotype distribution characteristics of 137 *S. suis* isolates collected from 12 regions of China. NT indicates non-typeable strains.

This study systematically analyzed the serotype distribution characteristics and regional differences of *S. suis* (Figure 3). In the eastern coastal provinces of China, there is a significant diversity of serotypes. In Guangdong, 9 serotypes, including serotypes 2, 5, 7, 8, 9, 16, and 33, and serotype NT were detected. In Jiangsu Province, 8 serotypes, including serotypes 2, 3, 4, 5, 7, 9, 18, and NT, were detected. In Zhejiang Province, 7 serotypes, including serotypes 2, 3, 4, 5, 7, 9, and serotype NT, were detected. The prevalence of Serotype 2 was high in Guangdong (11 cases), Guangxi (7 cases), and Anhui (6 cases). In contrast, 1–3 serotypes were detected in inland provinces, such as Hubei, Hunan, and Shaanxi. In Liaoning Province, only Serotype 1 (2 cases) was identified. This distribution pattern may be influenced by sample representation, geographic barriers, or ecological niche competition among dominant serotypes.

**Figure 4:**
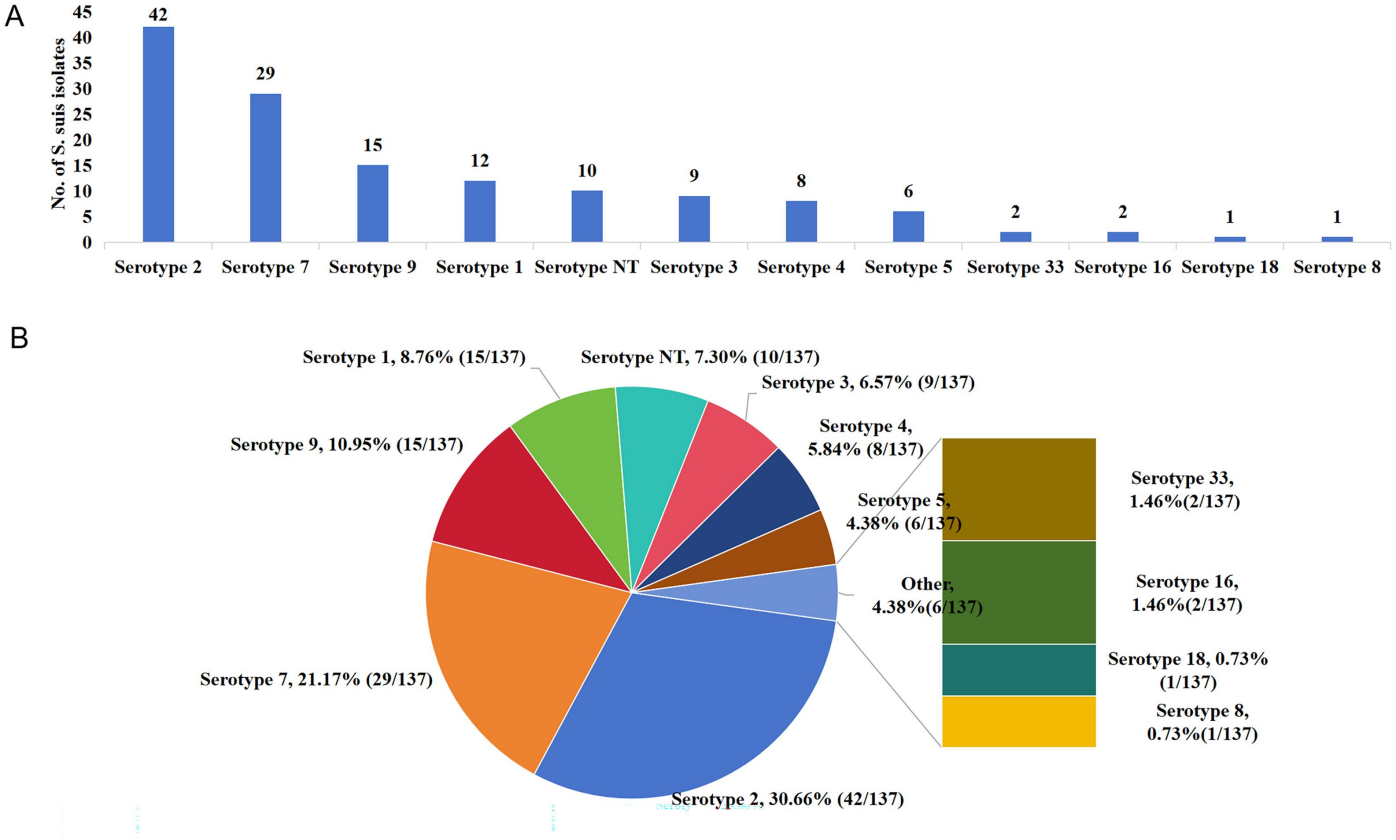
Serotype distribution patterns of 137 *S. suis* isolates. **(A)** Number of isolates corresponding to each serotype; **(B)** detailed breakdown of isolates by serotype.

Further analysis of the 137 isolates revealed that Serotype 2 presented the highest occurrence rate (*n* = 42; 30.66%), followed by Serotype 7 (*n* = 29; 21.17%), Serotype 9 (*n* = 15; 10.95%), Serotype 1 (*n* = 12; 8.76%), Serotype NT (*n* = 10; 7.30%), Serotype 3 (*n* = 9; 6.57%), Serotype 4 (*n* = 8; 5.84%), and Serotype 5 (*n* = 5; 4.38%). serotypes 33 (*n* = 2; 1.46%), serotype 16 (*n* = 2; 1.46%), serotype 8 (*n* = 1; 1.46%), and serotype 8 (*n* = 1; 1.46%) (Figures 4A, B).

### 3.3 Detection of virulence genes in *S. suis*

**Figure 5:**
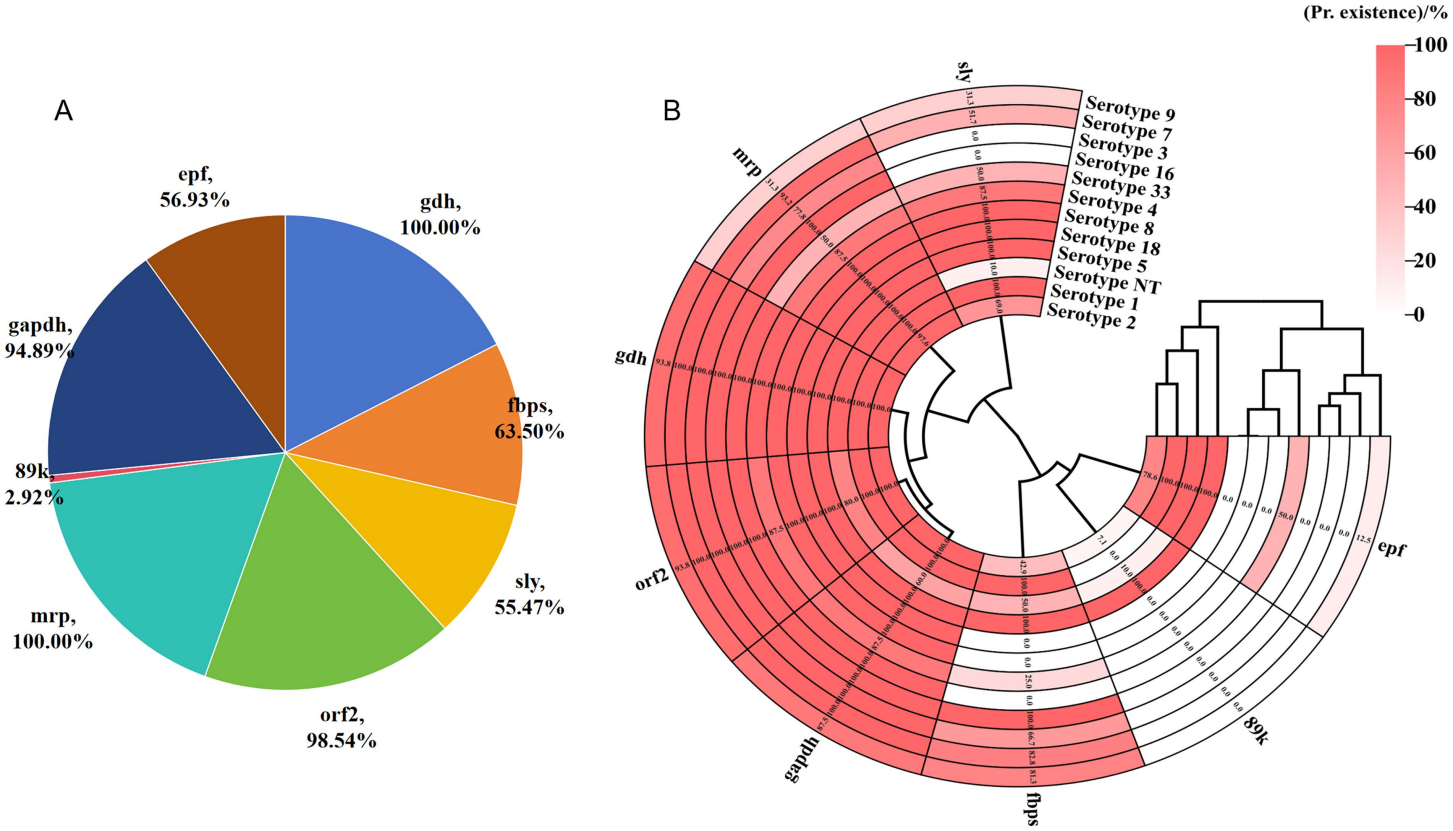
Relationships between serotypes and virulence genes in 137 *S. suis* isolates. **(A)** Positive counts of different virulence genes detected in the 137 isolates. **(B)** Heatmap showing the relationships between different serotypes and virulence genes among the isolates.

This study analyzed the associations between eight virulence genes (*gdh*, *fbps*, *sly*, *orf2*, *mrp*, *89 k*, *gapdh*, and *epf*) and serotypes in 137 *S. suis* isolates. The occurrence rates of *gdh*, *fbps*, *sly*, *orf2*, *mrp*, *89 k*, *gapdh*, and *epf* were 100% (137/137), 63.5% (87/137), 55.47% (76/137), 98.54% (135/137), 100% (137/137), 2.92% (4/137), 94.89% (130/137), and 56.93% (78/137), respectively (Figure 5A). All 12 isolates of Serotype 1 harbored *mrp*. Additionally, the other tested genes, with the exception of *89 k*, were detected in all the Serotype 1 isolates. The isolates of Serotype 2 and NT harbored all the tested genes. However, for serotypes 2 and NT, the incidence rates of 89 k were 4.76% (95% CI: 13.2–15.79%) and 10% (95% CI: 17.9–40.42%), respectively. Serotypes 4 and 7 did not harbor *89 k* or *epf*. The occurrence rates of *gdh* and *orf2* in serotypes 4 and 7 were 100% and ≥ 87%, respectively. The occurrence rates of *epf* in serotypes 5 and 7 were 20% (95% CI: 36.2–62.45%) and 0% (95% CI: 0–11.7%), respectively. The frequency of occurrence of most virulence factors, except for *sly*, *89 k*, and *epf*, was high in Serotype 7, with that of *gdh* and *orf2* reaching 100%. Serotype 9 did not harbor *89 k*. The occurrence rates of *sly*, *epf*, and *mrp* in Serotype 9 were ≤ 43.75%, with *epf* exhibiting a low occurrence rate (12.50, 95% CI: 35–36.02%). However, the occurrence rates of *gdh*, *fbps*, *gapdh*, and *orf2* in Serotype NT were in the range of 87.5–100%. Additionally, all Serotypes 8, 16, 18, and 33 harbored *fbps*, *orf2*, *mrp*, and *gapdh*. However, further validation is necessary, as the sample size for these isolates was small (< 3 isolates) (Figure 5B).

### 3.4 Animal experiment

**Figure 6:**
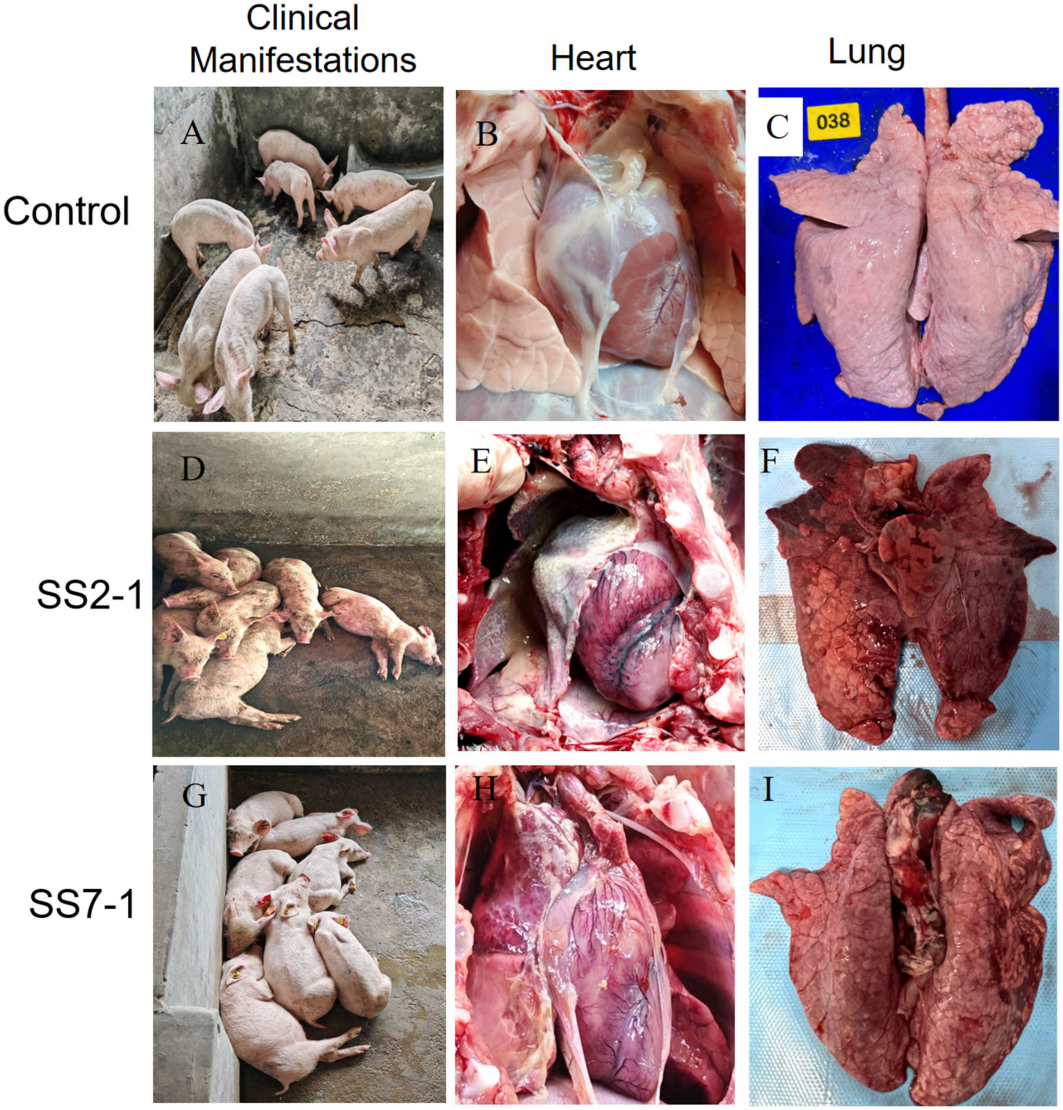
Clinical manifestations and post-mortem lesions in 42-day-old Landrace pigs after artificial infection. **(A–C)** Show the clinical status, heart, and lung lesions of pigs in the control group; **(D–F)** depict the clinical status, heart, and lung lesions in the SS2-challenged group; **(G–I)** illustrate the clinical status, heart, and lung lesions in the SS7-challenged group.

**Figure 7:**
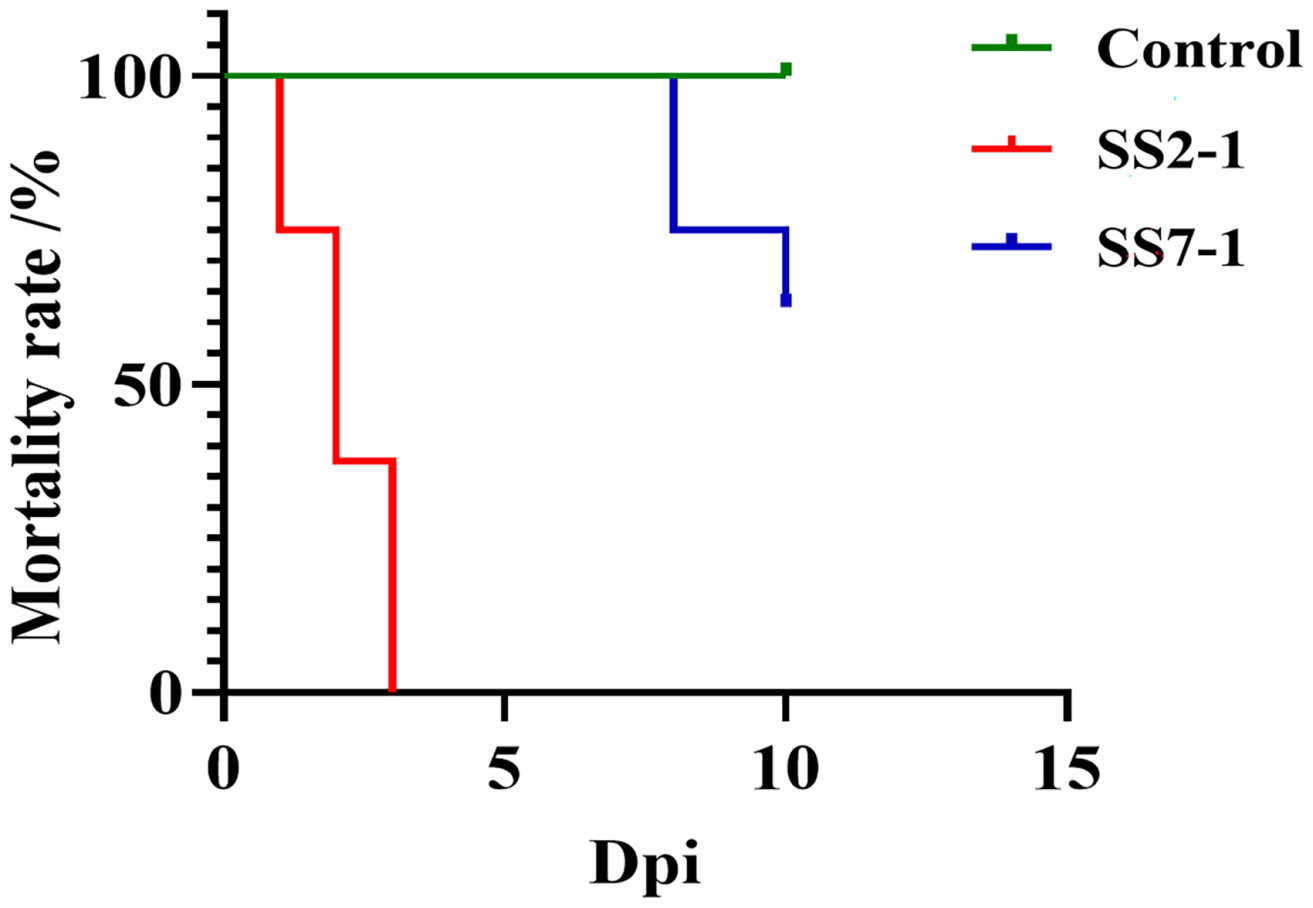
Survival curves of 42-day-old Landrace pigs after artificial infection with *S. suis.* The survival rates for the SS-2 and SS-7 challenge groups were 0 and 71.42%, respectively, whereas no deaths occurred in the control group.

To investigate the pathogenicity of the prevalent *S. suis* serotypes 2 and 7, strains SS2-1 and SS7-1 were selected for animal experimentation in 42-day-old, healthy Landrace pigs confirmed to be free of *S. suis* and other exogenous pathogens. The experimental results demonstrated that pigs in both the SS2-1 and SS7-1 challenge groups presented clinical signs such as lethargy, huddling, and a marked reduction or complete loss of appetite within 12 h post-infection (Figures 6D,G). Notably, all pigs in the SS2-1 group succumbed to acute infection by the fourth day, resulting in a mortality rate of 100% (8/8). In the SS7-1 group, no acute deaths occurred, but two pigs died on the 10th day, resulting in a mortality rate of 37.5% (3/8) (Figure 7). The surviving pigs were emaciated and exhibited slow growth, whereas no significant changes were detected in the control group (Figure 6A).

Necropsy was performed on pigs that died after infection. The results revealed the following: In the SS2-1 group, the deceased pigs exhibited typical acute fibrinous myocarditis, with yellow-brown flocculent exudates accumulating in the pericardial cavity (Figure 6E). The lungs exhibited diffuse congestion and edema, characterized by dark red congested areas along the margins of the lung lobes (Figure 6F). In the SS7-1 group, the pathological changes in the deceased pigs were similar to those observed in the SS2-1 group but were comparatively less severe. The principal findings included surface cardiac hemorrhage and enlargement, myocardial hemorrhage, and mild fibrinous myocarditis (Figure 6H). The lungs were enlarged and hemorrhagic, and a small quantity of serous or mildly fibrinous exudate was present within the thoracic cavity (Figure 6I). In contrast, no significant pathological alterations were observed in the control group during necropsy (Figures 6B,C).

**Figure 8:**
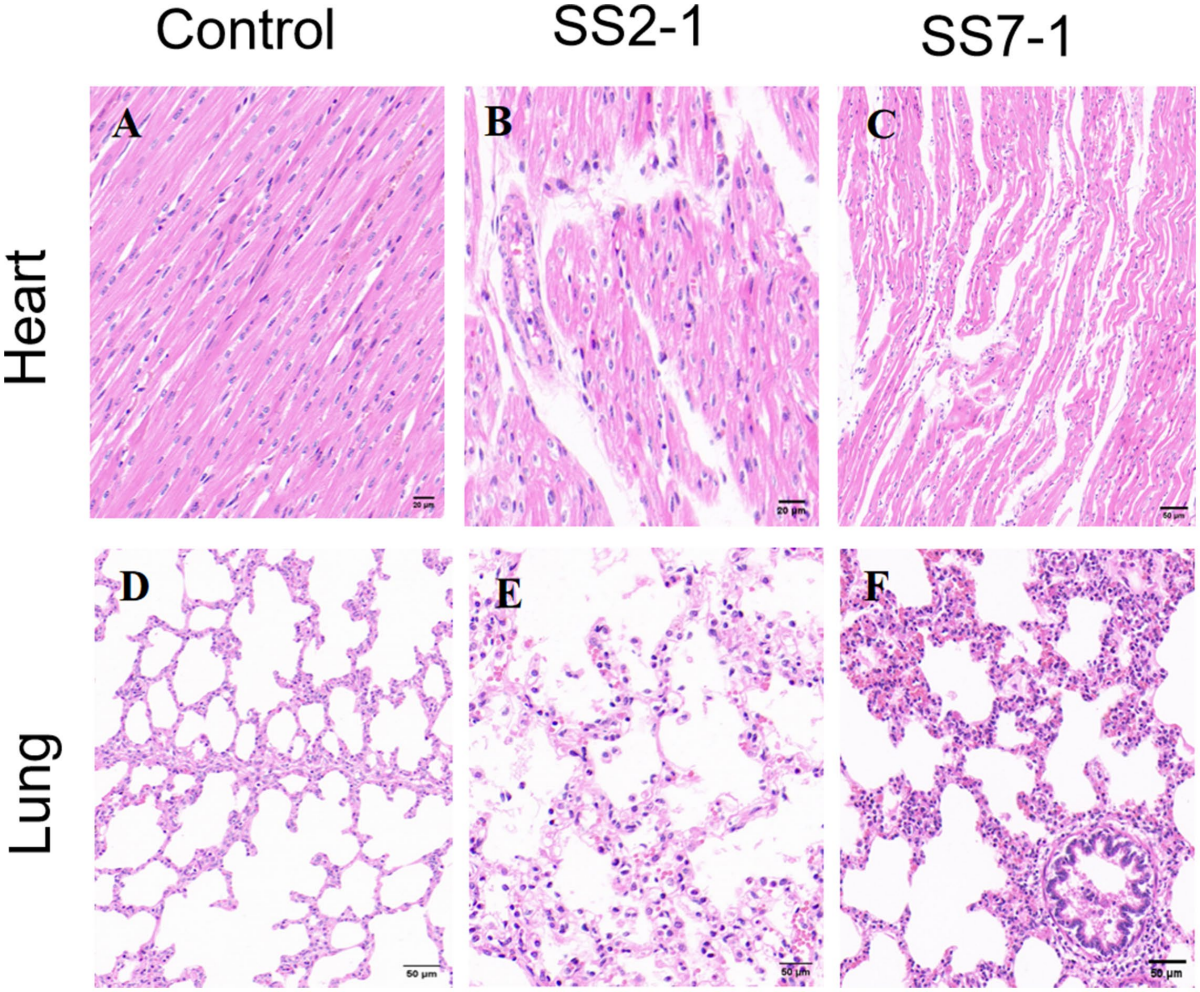
Histopathological changes in the heart and lungs of 42-day-old Landrace pigs after artificial infection. **(A–C)** Represent histopathological changes in the lungs of the control group, SS2-challenged group, and SS7-challenged group, respectively; **(D–F)** show histopathological changes in the hearts of the control group, SS2-challenged group, and SS7-challenged group, respectively.

Histopathological examination revealed that in both the SS2-1 and SS7-1 groups, myocardial cells exhibited disorganization, increased infiltration of inflammatory cells, interstitial edema, and erythrocyte exudation (Figures 8B,C). The alveolar structures were either disrupted or altered, with eosinophilic exudates and scattered erythrocyte leakage present within the alveolar cavities. Hemorrhage and enlargement were also prominent in the lung interstitium (Figures 8E,F). In contrast, no significant pathological alterations were observed in the control group (Figures 8A,D).

## 4 Discussion

*S. suis* is one of the most threatening bacterial infectious diseases in the global pig farming industry. Its typical clinical symptoms include purulent meningitis, septicemia, and polyarthritis, which impose substantial economic burdens on global swine production systems annually (Dutkiewicz et al., 2018). Epidemiological investigations have shown that in several regions, including Europe, North America, and Australia, the infection rate of *Streptococcus suis* has exceeded 90%. In China, the carrier rate of this pathogen on large-scale pig farms reportedly exceeds 40% (Segura et al., 2020). This phenomenon not only poses a serious threat to the health of pigs but also has a significant impact on the global agricultural economy. This study, which is based on cross-regional epidemiological surveys, revealed that the overall detection rate of *S. suis* in large-scale pig farms across 12 provinces in China reached 59.59%, which is markedly higher than the previou*sly* reported prevalence of 40.8% (Liu et al., 2023). Importantly, the findings also revealed the pathogen’s full-cycle infection capability, with positive cases identified across all age groups of pigs, underscoring its persistent and pervasive threat within pig farming systems. The infection rates in suckling piglets (<21 days old) and nursery pigs (21–70 days old) were 57.4 and 61.21%, respectively, which is consistent with previous reports (Correa-Fiz et al., 2020). Previous studies have indicated that *S. suis* infections can occur year-round, with higher isolation rates observed during summer months, likely due to increased humidity during this season (Zhang et al., 2019). However, this study revealed that infection rates were significantly higher in winter and spring than in summer and autumn, a finding that may be associated with regional factors.

In recent years, Chinese researchers have identified several novel variants, including serotypes 21/29, NCL21-NCL26, and Chz, expanding the known diversity of *S. suis* and underscoring the evolving complexity of its epidemiological landscape (Huang et al., 2019; Pan et al., 2015). Although the prevalence of *S. suis* serotypes and genotypes varies across geographic regions and time periods, serotype 2 remains one of the most prevalent serotypes globally. Moreover, the distribution of *S. suis* serotypes shows significant regional differences: In Spain, serotypes 2 (21.7%), 1 (21.3%), and 9 (19.3%) dominate (Petrocchi-Rilo et al., 2021), whereas in North America, serotypes 2 (24.3%) and 3 (21%) prevail. In Asia, serotype 2 accounts for the highest proportion (44.2%), with a detection rate of 34.08% in Jiangxi Province, China, whereas serotypes 3 and 4 account for 12.4 and 5.6%, respectively (Tan et al., 2021). Similarly, this investigation revealed that the distribution of *S. suis* serotypes in China displays pronounced regional characteristics. For example, multiple serotypes were found to coexist within individual regions along the eastern coast, such as Guangdong, Jiangsu, and Shandong. Notably, serotype NT was highly prevalent in Anhui, Guangxi, and Guangdong, accounting for 65.5% of all NT isolates—a pattern seldom reported in the literature. On a global scale, the predominant *S. suis* serotypes isolated from clinical pigs, in descending order of frequency, are serotypes 2, 9, 3, 1/2, and 7, with about 15% classified as NT (Goyette-Desjardins et al., 2014). In this study, serotypes NT and 2 were dominant, followed by serotype 7, which is partially consistent with global trends. However, the higher proportion of NT (21.01%) suggests that regional characteristics or differences in detection methods may influence serotype distribution patterns. The lungs serve as the core infection site (33.33–100%), with serotypes 2, 7, 9, and NT exhibiting multiorgan invasion capabilities, indicating strong tissue invasiveness, which exacerbates disease complexity and control challenges.

The virulence of *S. suis* is closely associated with its serotypes, with notable variations in virulence factors across different serotypes. To date, over 100 virulence-associated components have been identified, including hemolysins, adhesins, and proteases, which collectively contribute to the pathogen’s differential pathogenicity (Roodsant et al., 2021). Among these proteins, hemolysin *sly* (57 kDa) is recognized as a pivotal virulence factor capable of disrupting the blood–brain barrier, inhibiting complement-mediated bactericidal activity, and triggering robust inflammatory responses (Lin et al., 2019). Among the serotype 2 strains, *sly*-positive isolates were significantly correlated with increased pathogenic potential. Yin et al. (2016) reported the *89 k* pathogenicity island in serotype 2 *S. suis*, a genomic element strongly linked to severe outbreaks in Jiangsu and Sichuan and regarded as a major determinant of virulence. However, in the present study, the detection rate of the *89 k* pathogenicity island in serotype 2 strains was only 7.14% (2/28), and intriguingly, the gene was also detected in serotype 16 and NT strains, suggesting a broader distribution and variability than previou*sly* reported.

*mrp*, *epf*, *fbps*, and *sly* are recognized as key pathogenic marker genes of *S. suis* serotype 2 (Dong et al., 2015). Previous reports have indicated the universal presence of the *sly* gene across isolates, whereas the carriage rates of *mrp* and *epf* were notably lower, at 33 and 4%, respectively, which contrasts markedly with those of clinical isolates from North America and Europe, where the carriage rates of *mrp* and *epf* can reach 92 and 31%, respectively (Fittipaldi et al., 2009; Kim et al., 2010). In the present study, however, the carriage rates of *sly*, *mrp*, and *epf* in serotype 2 isolates were 92.85% (26/28), 96.43% (27/28), and 78.57% (22/28), respectively—levels comparable to those reported for clinical isolates from North America and Europe. Additionally, the *mrp* gene was highly prevalent in serotypes 1, 3, 4, 5, 7, 8, 9, 16, 18, 33, and NT, indicating that *mrp* might be a key virulence factor of porcine-derived *S. suis*.

The *gdh* gene of *S. suis* is a specific protein that can serve as a marker antigen for detection. About 305 porcine serum samples were found to have a *gdh* seropositivity rate of 73.1%, suggesting its potential application in diagnosing *S. suis* infections (Xia et al., 2017). This study further confirmed this finding: among 137 *S. suis* isolates, the carriage rate of the *gdh* gene was 96.35% (132/137). Except for serotype NT, where the carriage rate was 82.76%, all other serotypes carried the *gdh* gene at 100%. These findings indicate that the *gdh* gene is also a key virulence factor of porcine-derived *S. suis*, and studies on *gdh* gene deletion provide important insights for vaccine development.

gapdh is an important virulence factor closely linked to the bacterial adhesion process. Studies have demonstrated that deletion of the *gapdh* gene significantly impairs the ability of bacteria to adhere to host cells (Brassard et al., 2004). Research further indicates that the *gapdh* gene is widely distributed among various streptococcal species, including serotypes 2, 7, and 9 of *S. suis*. Only a few nonpathogenic strains lack this gene, underscoring its universality and importance (Wang et al., 2021). This study also supports this view, with the carriage rate of the *gapdh* gene being 89.78% (123/137). Serotypes 1, 2, 3, 7, 8, 16, 18, and 33 carried it at 100%, whereas serotypes 4, 5, 9, and NT had carriage rates as high as 87.5%. Thus, regardless of whether the strain is low or highly virulent, this gene is universally present. The *orf2* gene is closely related to the virulence of *Streptococcus suis*, and it is present in at least 78.3% of *Streptococcus suis* isolates (Zhao et al., 2025). This study revealed that the carriage rate of *orf2* in 137 isolates was as high as 93%, and *orf2* is widely present in different serotypes, suggesting its universality. However, its function and impact on virulence need to be comprehensively evaluated in combination with other factors.

Currently, limited research has been conducted on the pathogenicity of *S. suis* serotypes 2 and 7 in pigs, with the majority of existing studies being based on mouse models. Evidence indicates that serotype 2 is the most virulent and is capable of inducing acute mortality in pigs through septicemia and polyserositis (Lun et al., 2007). The present study corroborates this finding, which may be attributed to the high-frequency carriage of key virulence genes, including *gdh*, *fbps*, *sly*, *orf2*, *mrp*, and *gapdh*. In challenge experiments with serotype 7 in 42-day-old Landrace pigs, although no acute deaths were observed, two pigs died on the 10th day post-infection, both of which presented with leg arthritis and typical polyserositis lesions. This result is consistent with the experimental findings of Boetner AG et al., who used serotype 7 to infect 7-day-old piglets, indicating that serotype 7 has some pathogenicity in piglets of this age group (Boetner et al., 1987). Additionally, 42-day-old Landrace pigs infected with serotypes 2 and 7 presented obvious pneumonia and myocarditis symptoms, suggesting that the lungs and heart may be the primary target organs of these two serotypes.

In summary, this study revealed that the overall infection rate is markedly higher than previou*sly* reported, with pigs of all age groups demonstrating susceptibility and no evident seasonal variation. Serotypes NT and 2 emerged as the predominant strains, followed by serotype 7. While this distribution trend is partially consistent with global patterns, the elevated prevalence of serotype NT indicates that regional factors or methodological differences in detection may contribute to variations in serotype distribution. Notably, the carriage rates of virulence genes varied significantly across serotypes, with *gdh*, *fbps*, *sly*, *orf2*, *mrp*, and *gapdh* being widely detected, whereas *89 k* and *epf* were found at lower frequencies. Moreover, both serotypes 2 and 7 can cause clinical symptoms similar to those of *S. suis* disease, but serotype 2 is significantly more pathogenic than serotype 7. The absence of antimicrobial resistance profiling for the collected *S. suis* isolates constitutes a limitation of this work. Subsequent research is planned to explore this area comprehensively.

## 5 Conclusion

In this study, we investigated the prevalence of *S. suis* in 89 large-scale pig farms in 12 provinces in the western region of China and analyzed the serotypes and presence of virulence genes of the isolates as well as the pathogenicity of serotypes 2 and 7. The results of this study provide important baseline information on the serotype characteristics and virulence genes of *S. suis* and the pathogenicity of epidemic strains in China, which is highly important for understanding its epidemiological characteristics and the development of vaccines used to prevent *Streptococcus suis* infection in pigs.

## Data Availability

The original contributions presented in the study are included in the article/supplementary material, further inquiries can be directed to the corresponding author.

## References

[ref1] Bamphensin N. Chopjitt P. Hatrongjit R. Boueroy P. Fittipaldi N. Gottschalk M. . (2021). Non-penicillin-susceptible *streptococcus suis* isolated from humans. Pathogens. 10:1178. doi: 10.3390/pathogens10091178, 34578210 PMC8471365

[ref2] Boetner A. G. Binder M. Bille-Hansen V. (1987). *Streptococcus suis* infections in Danish pigs and experimental infection with *Streptococcus suis* serotype 7. Acta Pathol. Microbiol. Immunol. Scand. B 95, 233–239. doi: 10.1111/j.1699-0463.1987.tb03118.x, 3673579

[ref3] Brassard J. Gottschalk M. Quessy S. (2004). Cloning and purification of the *streptococcus suis* serotype 2 glyceraldehyde-3-phosphate dehydrogenase and its involvement as an adhesin. Vet. Microbiol. 102, 87–94. doi: 10.1016/j.vetmic.2004.05.008, 15288930

[ref4] Correa-Fiz F. Neila-Ibanez C. Lopez-Soria S. Napp S. Martinez B. Sobrevia L. . (2020). Feed additives for the control of post-weaning *streptococcus suis* disease and the effect on the faecal and nasal microbiota. Sci. Rep. 10:20354. doi: 10.1038/s41598-020-77313-6, 33230191 PMC7683732

[ref5] Devriese L. A. Haesebrouck F. (1992). *Streptococcus suis* infections in horses and cats. Vet. Rec. 130:380. doi: 10.1136/vr.130.17.380, 1604787

[ref6] Dong W. Ma J. Zhu Y. Zhu J. Yuan L. Wang Y. . (2015). Virulence genotyping and population analysis of *streptococcus suis* serotype 2 isolates from China. Infect. Genet. Evol. 36, 483–489. doi: 10.1016/j.meegid.2015.08.021, 26303637

[ref9001] Dutkiewicz J. Zajac V. Sroka J. Wasinski B. Cisak E. Sawczyn A. . (2018). Streptococcus suis: a re-emerging pathogen associated with occupational exposure to pigs or pork products. Part ii - pathogenesis. Ann. Agr. Env. Med. 25, 186–203. doi: 10.26444/aaem/85651 29575852

[ref7] Fittipaldi N. Fuller T. E. Teel J. F. Wilson T. L. Wolfram T. J. Lowery D. E. . (2009). Serotype distribution and production of muramidase-released protein, extracellular factor and suilysin by field strains of *streptococcus suis* isolated in the United States. Vet. Microbiol. 139, 310–317. doi: 10.1016/j.vetmic.2009.06.024 19596529

[ref8] Gottschalk M. Lebrun A. Wisselink H. Dubreuil J. D. Smith H. Vecht U. (1998). Production of virulence-related proteins by Canadian strains of *Streptococcus suis* capsular type 2. Can. J. Vet. Res. 62, 75–79, 9442945 PMC1189447

[ref9] Goyette-Desjardins G. Auger J. P. Xu J. Segura M. Gottschalk M. (2014). *Streptococcus suis*, an important pig pathogen and emerging zoonotic agent-an update on the worldwide distribution based on serotyping and sequence typing. Emerg. Microbes Infect. 3:e45. doi: 10.1038/emi.2014.45, 26038745 PMC4078792

[ref10] Haas B. Grenier D. (2018). Understanding the virulence of *streptococcus suis*: a veterinary, medical, and economic challenge. Med. Mal. Infect. 48, 159–166. doi: 10.1016/j.medmal.2017.10.001, 29122409

[ref11] Huang J. Liu X. Chen H. Chen L. Gao X. Pan Z. . (2019). Identification of six novel capsular polysaccharide loci (ncl) from *streptococcus suis* multidrug resistant non-typeable strains and the pathogenic characteristic of strains carrying new ncls. Transbound. Emerg. Dis. 66, 995–1003. doi: 10.1111/tbed.13123, 30676694

[ref12] Ju A. Wang C. Zheng F. Pan X. Dong Y. Ge J. . (2008). Study on molecular epidemiology of major pathgenic *Streptococcus suis* serotypes in middle part of Jiangsu province. Chin. Epidemiol. 29, 151–154. 18686855

[ref9002] Kerdsin A. Akeda Y. Hatrongjit R. Detchawna U. Sekizaki T. Hamada S. . (2014). Streptococcus suis serotyping by a new multiplex pcr. J. Med. Microbiol. 63:824–830. doi: 10.1099/jmm.0.069757-0 24696517

[ref9003] Kerdsin A. Dejsirilert S. Akeda Y. Sekizaki T. Hamada S. Gottschalk M. . (2012). Fifteen streptococcus suis serotypes identified by multiplex pcr. J. Med. Microbiol. 61:1669–1672. doi: 10.1099/jmm.0.048587-0 22918870

[ref13] Kim D. Han K. Oh Y. Kim C. H. Kang I. Lee J. . (2010). Distribution of capsular serotypes and virulence markers of *streptococcus suis* isolated from pigs with polyserositis in Korea. Can. J. Vet. Res. 74, 314–316. 21197232 PMC2949345

[ref14] King S. J. Heath P. J. Luque I. Tarradas C. Dowson C. G. Whatmore A. M. (2001). Distribution and genetic diversity of suilysin in *streptococcus suis* isolated from different diseases of pigs and characterization of the genetic basis of suilysin absence. Infect. Immun. 69, 7572–7582. doi: 10.1128/IAI.69.12.7572-7582.2001, 11705935 PMC98849

[ref15] Lin L. Xu L. Lv W. Han L. Xiang Y. Fu L. . (2019). An nlrp3 inflammasome-triggered cytokine storm contributes to streptococcal toxic shock-like syndrome (stsls). PLoS Pathog. 15:e1007795. doi: 10.1371/journal.ppat.1007795, 31170267 PMC6553798

[ref16] Liu P. Zhang Y. Tang H. Wang Y. Sun X. (2023). Prevalence of *streptococcus suis* in pigs in China during 2000-2021: a systematic review and meta-analysis. One Health. 16:100513. doi: 10.1016/j.onehlt.2023.100513, 37363255 PMC10288055

[ref17] Liu Z. Zheng H. Gottschalk M. Bai X. Lan R. Ji S. . (2013). Development of multiplex pcr assays for the identification of the 33 serotypes of *streptococcus suis*. PLoS One 8:e72070. doi: 10.1371/journal.pone.0072070, 23951285 PMC3739753

[ref18] Lun Z. R. Wang Q. P. Chen X. G. Li A. X. Zhu X. Q. (2007). *Streptococcus suis*: an emerging zoonotic pathogen. Lancet Infect. Dis. 7, 201–209. doi: 10.1016/S1473-3099(07)70001-4, 17317601

[ref19] Luque I. Tarradas C. Arenas A. Maldonado A. Astorga R. Perea A. (1998). *Streptococcus suis* serotypes associated with different disease conditions in pigs. Vet. Rec. 142, 726–727. doi: 10.1136/vr.142.26.726, 9682433

[ref20] Mi K. Li M. Sun L. Hou Y. Zhou K. Hao H. . (2021). Determination of susceptibility breakpoint for cefquinome against *streptococcus suis* in pigs. Antibiotics-Basel. 10:958. doi: 10.3390/antibiotics10080958, 34439008 PMC8389024

[ref21] Nomoto R. Maruyama F. Ishida S. Tohya M. Sekizaki T. Osawa R. (2015). Reappraisal of the taxonomy of *streptococcus suis* serotypes 20, 22 and 26: *streptococcus parasuis* sp. nov. Int. J. Syst. Evol. Microbiol. 65, 438–443. doi: 10.1099/ijs.0.067116-0, 25385995

[ref22] Pan Z. Ma J. Dong W. Song W. Wang K. Lu C. . (2015). Novel variant serotype of *streptococcus suis* isolated from piglets with meningitis. Appl. Environ. Microbiol. 81, 976–985. doi: 10.1128/AEM.02962-14, 25416757 PMC4292476

[ref23] Pan J. Zhang H. Hen B. Zhou M. Wang Z. Xu G. (2020). Isolation, identification and pathogenicity of *Streptococcus suis* type 2. China J. Vet. Drug. 54, 14–19. doi: 10.11751/ISSN.1002-1280.2020.08.03

[ref24] Petrocchi-Rilo M. Martinez-Martinez S. Aguaron-Turrientes A. Roca-Martinez E. Garcia-Iglesias M. J. Perez-Fernandez E. . (2021). Anatomical site, typing, virulence gene profiling, antimicrobial susceptibility and resistance genes of *streptococcus suis* isolates recovered from pigs in Spain. Antibiotics-Basel. 10:707. doi: 10.3390/antibiotics10060707, 34208248 PMC8230935

[ref25] Roodsant T. J. Van Der Putten B. Tamminga S. M. Schultsz C. Van Der Ark K. (2021). Identification of *streptococcus suis* putative zoonotic virulence factors: a systematic review and genomic meta-analysis. Virulence 12, 2787–2797. doi: 10.1080/21505594.2021.1985760, 34666617 PMC8632099

[ref26] Salasia S. I. Lammler C. Devriese L. A. (1994). Serotypes and putative virulence markers of *streptococcus suis* isolates from cats and dogs. Res. Vet. Sci. 57, 259–261. doi: 10.1016/0034-5288(94)90070-1, 7817019

[ref27] Silva L. M. Baums C. G. Rehm T. Wisselink H. J. Goethe R. Valentin-Weigand P. (2006). Virulence-associated gene profiling of *streptococcus suis* isolates by PCR. Vet. Microbiol. 115, 117–127. doi: 10.1016/j.vetmic.2005.12.013, 16431041

[ref9004] Segura M. Aragon V. Brockmeier S. L. Gebhart C. Greeff A. Kerdsin A. . (2020). Update on streptococcus suis research and prevention in the era of antimicrobial restriction: 4th international workshop on s. Suis. Pathogens. 9. doi: 10.3390/pathogens9050374 PMC7281350 32422856

[ref9005] Smith H. E. Veenbergen V. van der Velde J. Damman M. Wisselink H. J. Smits M. A. (1999). The cps genes of streptococcus suis serotypes 1, 2, and 9: development of rapid serotype-specific pcr assays. J. Clin. Microbiol. 37, 3146–3152. doi: 10.1128/JCM.37.10.3146-3152.1999 PMC85514 10488168

[ref28] Tan M. F. Tan J. Zeng Y. B. Li H. Q. Yang Q. Zhou R. (2021). Antimicrobial resistance phenotypes and genotypes of *streptococcus suis* isolated from clinically healthy pigs from 2017 to 2019 in Jiangxi province, China. J. Appl. Microbiol. 130, 797–806. doi: 10.1111/jam.14831, 32881196

[ref29] Vecht U. Wisselink H. J. van Dijk J. E. Smith H. E. (1992). Virulence of *streptococcus suis* type 2 strains in newborn germfree pigs depends on phenotype. Infect. Immun. 60, 550–556. doi: 10.1128/iai.60.2.550-556.1992, 1730489 PMC257663

[ref30] Wang Z. Guo M. Kong L. Gao Y. Ma J. Cheng Y. . (2021). Tlr4 agonist combined with trivalent protein joints of *streptococcus suis* provides immunological protection in animals. Vaccine 9:958. doi: 10.3390/vaccines9020184, 33671673 PMC7926372

[ref31] Wisselink H. J. Reek F. H. Vecht U. Stockhofe-Zurwieden N. Smits M. A. Smith H. E. (1999). Detection of virulent strains of *streptococcus suis* type 2 and highly virulent strains of *streptococcus suis* type 1 in tonsillar specimens of pigs by PCR. Vet. Microbiol. 67, 143–157. doi: 10.1016/s0378-1135(99)00036-x, 10414368

[ref32] Wisselink H. J. Smith H. E. Stockhofe-Zurwieden N. Peperkamp K. Vecht U. (2000). Distribution of capsular types and production of muramidase-released protein (mrp) and extracellular factor (ef) of *streptococcus suis* strains isolated from diseased pigs in seven european countries. Vet. Microbiol. 74, 237–248. doi: 10.1016/s0378-1135(00)00188-7, 10808092

[ref33] Xia X. J. Wang L. Shen Z. Q. Qin W. Hu J. Jiang S. J. . (2017). Development of an indirect dot-ppa-elisa using glutamate dehydrogenase as a diagnostic antigen for the rapid and specific detection of streptococcus suis and its application to clinical specimens. Antonie Van Leeuwenhoek 110, 585–592. doi: 10.1007/s10482-016-0825-z, 28058577

[ref34] Yin S. Li M. Rao X. Yao X. Zhong Q. Wang M. . (2016). Subtilisin-like protease-1 secreted through type iv secretion system contributes to high virulence of *streptococcus suis* 2. Sci. Rep. 6:27369. doi: 10.1038/srep27369, 27270879 PMC4897608

[ref35] Zhang B. Ku X. Yu X. Sun Q. Wu H. Chen F. . (2019). Prevalence and antimicrobial susceptibilities of bacterial pathogens in chinese pig farms from 2013 to 2017. Sci. Rep. 9:9908. doi: 10.1038/s41598-019-45482-8, 31289289 PMC6616368

[ref36] Zhao X. Han S. Zhang F. Cui L. Ji G. Wang S. . (2025). Identification and characterization of *streptococcus suis* strains isolated from eastern China swine farms, 2021-2023. Sci. Rep. 15:5677. doi: 10.1038/s41598-025-90308-5, 39955355 PMC11829963

